# Why Do Some Employees Fall into and Fail to Exit a Job-Lock Situation?

**DOI:** 10.1155/2013/839349

**Published:** 2013-05-07

**Authors:** Anna Huysse-Gaytandjieva, Wim Groot, Milena Pavlova

**Affiliations:** ^1^Department of Health Services Research, CAPHRI, Maastricht University Medical Center, Faculty of Health, Medicine and Life Sciences, Maastricht University, Duboisdomein 30, 6229 Maastricht, The Netherlands; ^2^Psychotherapie Praktijk Limburg, Valkenburgerweg 95, 6321 GC Wijlre, The Netherlands; ^3^Top Institute for Evidence-Based Education Research (TIER), Maastricht University, Kapoenstraat 2, 6211 KR Maastricht, The Netherlands

## Abstract

Previous studies have paid little attention to the employees' ability to exit a job-lock situation and factors that determine this ability. It remains unclear why some employees who experience job lock are able to exit this state while others remain in job lock. We use longitudinal data to identify employees who have fallen in the state of job lock and their subsequent behavior—exiting or remaining in job lock. By use of a first-order Markov transition models, we analyze the relevance of sociodemographic features, employment, occupational, sectoral, and contextual factors, as well as personality characteristics in explaining the transition or its absence. Overall the results show that both demographic factors and work-related aspects increase the likelihood that an employee enters the long-term job lock state (especially for older, married, full-time employed, those in a craft occupation and governmental sector, and in a region with high unemployment). Mental health problems and personality characteristics (low peak-end self-esteem and decisional procrastination) have a significant effect on the probability to stay in long-term job lock. On the contrary, having a managerial, service, or associate occupation, working in the private sector, and having promotion opportunities increase the chance of an exit from the state of job lock.

## 1. Introduction

The desire to adapt to feelings of dissatisfaction is natural. Dissatisfied employees are likely to try to reduce their job dissatisfaction and work-related stress by adjusting to their current job or by changing jobs [1–3]. However, some employees fail to adapt to job dissatisfaction. These employees stay dissatisfied even though some of them may exhibit adaptive behavior. When employees are unable to adapt and remain in their unsatisfactory work situation in the long run, they can fall into job lock (become stuck in their job). The work performance of these employees may be reduced, if prolonged dissatisfaction leads to a negative attitude towards their work and withdrawal behavior [4], which can bring extra costs to the employing organization.

Various studies in the fields of economics and psychology have investigated the phenomenon of job lock and its determinants following the perspective of their own field (some examples include [5–8]). The combination of determinants proposed in the economics and psychology literature is found to have an important role in better understanding why some employees are in a job-lock situation [9].

Nevertheless, both economics and psychology studies have paid little attention to the employees' ability to exit a job-lock situation and factors that determine this ability. It remains unclear why some employees who experience job lock are able to exit this state while others remain in job lock. Also, for those employees who leave the state of job lock, it may be asked what the mechanisms are by which this happens—do they adjust by becoming satisfied, do they use mobility as a way of dealing with dissatisfaction, or a combination of both? The answers to these questions are important in developing interventions to assist employees to reduce work stress and successfully adapt to job dissatisfaction.

The aim of this paper is to investigate the process of transition from a job-lock situation (i.e., being dissatisfied with the job but remaining in the same job) to other states, for example, adjusting and becoming satisfied in the same job (immobile and job satisfied), changing jobs and becoming satisfied (mobile and job satisfied), or changing jobs but again become dissatisfied with the new job (mobile and job dissatisfied).

We compare those in job lock, who fail to make a transition, to those who experience one of the three transitions described previously. Also, we compare the transition processes *from* the job-lock state with the transition process *to* the job-lock state.

We combine insights from both economics and psychology studies to identify a set of possible transition determinants. In particular, based on Huysse-Gaytandjieva et al. [9], we study the relevance not only of sociodemographic, employment, occupational, sectoral, and contextual factors but also of personality characteristics in explaining the transition or its absence. With regard to personality characteristics, we include an indicator of self-esteem as a key characteristic of personality in relation to job lock [9]. In contrast to previous studies, we also include an indicator of procrastination, which is seen as a consequence of preexisting personality characteristics [10].

We use data from the British Household Panel Survey [11]. The BHPS is longitudinal data that allow us to identify employees who have fallen in the state of job lock and their subsequent behavior—exiting or remaining in a job lock. The dataset also provides indicators of both procrastination and self-esteem, in addition to other relevant factors mentioned previously, which allows studying the joint effect of all groups of factors.

Section 2 provides background information outlining the relation of demographics, work-related factors, and in particular personality characteristics (procrastination and self-esteem) to job lock. The subsequent sections present our research methods and results of our analysis. The paper concludes by a discussion on the relevance of our findings and suggestions for management and research.

## 2. Long-Term Adaptation to Job Dissatisfaction

Review studies in the area of economics and psychology [3, 12–15] suggest that there are two broad groups of factors that potentially determine the state of job dissatisfaction, absence of turnover (job immobility), and/or job lock (or being “stuck” at work). These groups are employee's personal characteristics (sociodemographics and personality attributes) as well as work-related factors. The study of Huysse-Gaytandjieva et al. [9], which uses an interdisciplinary approach of labor economics and social psychology to study the state of job lock, combines indicators of these factors to explain why employees differ in the way they adjust to job dissatisfaction. Detailed elaboration on the relation between the group of factors and the job-lock state can be found in the same study [9]. In short, the study provides evidence that both groups of factors (personal characteristics and work-related factors) can jointly predict the state of job lock. Among employees who report job dissatisfaction for two subsequent years, those who are young, with low self-esteem, without an employer pension scheme, and working for a short time with the employer are more likely to remain immobile even though they are dissatisfied with their job (i.e., are in a job-lock situation). The study concludes that the adaptation to job dissatisfaction could be better understood if personality attributes (such as self-esteem) are included in the analysis [9]. The analysis in this paper goes one step further and investigates the effect of personal and work-related factors on falling into a state of long-term job lock (i.e., the inability to exit the job-lock state). In addition to self-esteem, we also include procrastination as a personality attribute that can explain long-term job lock. In the following we specifically discuss the importance of self-esteem and procrastination in relation to long-term job lock, which is seen as a self-regulation failure, that is, failure to adapt.

### 2.1. Self-Esteem and Procrastination in relation to Long-Term Job Lock

Successful adaptation to job dissatisfaction is seen as an alleviation of the job dissatisfaction level as a result of engaging in some adjusting mechanism [16]. The more an employee becomes dissatisfied at work, the more likely he/she is to engage in impulsive reactive behaviors (quitting, disengaging, and retaliation), rather than adaptive behaviors (problem solving or adjusting expectations) [17].

There are various personality characteristics related to responses to dissatisfaction and adaptation. The value of self-esteem in the adaptation process is in particular emphasized in the literature [18–22]. Here, we concentrate on the role of positive feelings of self-worth (secure high self-esteem) in prolonged job lock (i.e., the failure to exit a job-lock state).

Self-esteem is shown to be a personality characteristic that protects people against stressful consequences [23]. Thus, self-esteem is a personality characteristic that prevents people from experiencing long-term feelings of dissatisfaction. People with high self-esteem engage in positive, active attempts to cope with stressors [24, 25]. Numerous studies relate low self-esteem to adjustment problems [26–29]. Further, the ease of movement to another job is related to a subjective perception of available opportunities [30] and self-esteem [31, 32]. Those with low self-esteem tend to become preoccupied with distress emotions and are more likely to disengage from their goals when under stress. Low self-esteem not only strengthens negative feelings, but also undermines the ability to adequately cope with these feelings [33].

Much of the research about the relationship between self-esteem and health appears to have been done in terms of the influence of self-esteem on health-related behaviors. From the other side, in a review of the self-esteem literature, Baumeister et al. [34] conclude that the benefits of high self-esteem fall into two categories: enhanced initiative and pleasant feelings. They deduce that self-esteem has little association with health behavior. Additionally, the well-established relationship between self-esteem and psychological well-being (e.g., depression, social anxiety, and loneliness) [35] may be an important factor in understanding the relationship between self-esteem and health.

Furthermore, a negative self-image is important for the occurrence of procrastination [36]. Procrastinators show significantly lower self-esteem than nonprocrastinators [37]. Procrastination can be described as avoidance behavior, as “the avoidance of execution of an intended action” [38]. Actions (e.g., making decisions, searching for another job) have a cognitive importance for the individual but they may bring unpleasant feelings which cause an approach-avoidance conflict. This responds to the Janis and Mann's conflict model of decision making [39] which differentiates adaptive and nonadaptive patterns of coping with challenge. One of the nonadaptive patterns is defensive avoidance. It responds to the situation when any available alternative is perceived as risky and the decision maker is not hopeful to find a better solution. As a consequence, an escape from making a decision by procrastinating is following. Two types of procrastination are distinguished: decisional, the purposive delay in making decisions, and behavioral, delaying tasks to protect oneself due to a vulnerable self-esteem [37]. Decisional and behavioral procrastination are significantly correlated with each other [40]. Chronic procrastinators compared to nonprocrastinators have high rates of anger, hostility, depression, and actively self-handicap their task performance [40].

Procrastination may become dysfunctional when people frequently habitually delay to begin or complete tasks [40]. It may also affect job mobility, that is, delay mobility. In particular, procrastinators view their self-worth as determined by their ability [41]. When people feel incapable of making decisions, they might be more inclined to delay making a choice. Procrastinators, when they are dissatisfied with their job, might postpone job-seeking activities. This might be due to lower levels of self-determined job seeking motivation [42] or fear of failure if they try to search for a new job [43, 44]. Consequently, this will lead to a delay or lack of job mobility (job turnover). Thus, in the long run, low self-esteem has a strong relationship with procrastination, distress, and psychological health. Further, procrastinators fail to react based on their intentions [45]—job search intention and job search behavior. However, based on the action phases model of Gollwitzer [46], it is important to distinguish a predecisional phase (taking into consideration how to achieve goals) and a post-decisional phase (choice of behavior). In this study, we are concerned with the predecisional procrastination phase. The cross-cultural study by Ferrari et al. [47] that investigates the global rates of procrastination in United States, United Kingdom, and Australia concludes that “procrastination is widespread in westernized, individualistic, English-speaking countries.”

Given the aforementioned, additionally to self-esteem, in this study, we include procrastination as a variable that can be seen as a consequence of preexisting personality characteristics as self-esteem and as “an agent for bringing about adverse consequences of its own right” [10]. With regard to personality characteristics, we argue that people who are in job lock may perceive themselves incapable of making decisions about adaptation to job dissatisfaction and may postpone making such decisions, or be incapable of changing their unsatisfactory work situation and choose to remain in the same state [41]. Thus, both procrastination, that is, the inability to make timely decisions [39, 48, 49], and vulnerable self-esteem may play a role in explaining why some employees fall into or fail to exit a job-lock situation. While the importance of including self-esteem in job-lock models has been shown in previous research [9], the relevance of procrastination has not been studied yet. Moreover, little is known about decisional procrastination and its personality correlates [50, 51]. Various disciplines have shown an interest in and studied procrastination. Economists have related procrastination to a lack of retirement savings behavior [52], while psychologists relate it to planning personal health issues [53–55], regulation [56], and job seeking [42]. To our knowledge, till now, no study has considered procrastination directly in relation to job-to-job mobility, which is done in this paper. On the basis of presented theoretical insights, we expect that employees with low self-esteem, who procrastinate and have mental health problems, are more likely to enter long-term job lock compared to those who are with high self-esteem, do not procrastinate, and do not have mental health problems.

## 3. Materials and Methods

### 3.1. Data

We use data from the British Household Panel Survey (BHPS). The BHPS is an annual longitudinal survey based on a nationally representative sample of about 10,000 adults in Great Britain. Individuals are interviewed in successive waves. Details about the survey can be found in Taylor et al. [11]. Due to the change in the job satisfaction question in 1997, we only use data for the period 1991 to 1996 to assure the comparability across years. We include in our sample all men and women in the BHPS who are employed for at least three consecutive years (three survey waves) in the period mentioned previously, and who report dissatisfaction with their job for at least two subsequent years (2949 respondents in total). Unemployed and self-employed individuals are excluded.

### 3.2. Transition Models

To construct the models for our analysis, we use data related to job dissatisfaction and job immobility provided by the BHPS dataset. In particular, the job dissatisfaction variable for our analysis is derived from the BHPS variable that indicates the overall job satisfaction of a respondent measured on a seven-point Likert scale. Thus, we construct a dummy job-dissatisfaction variable for each year (0 = job satisfaction; 1 = job dissatisfaction). The category “neither satisfied nor dissatisfied” is seen as indicative of not being all that satisfied with the job [57] and, hence, it is included in the “dissatisfied” category.

We derive the job immobility variable from the BHPS variable that indicates tenure: “What was the date you started working in your present position, by that I mean the beginning of your current spell of the job you are doing now for your present employer?”.   If in a given year, tenure is greater or equal to one year, job immobility is coded with zero, and if tenure is less than one year, job immobility is coded with one. Thus, a dummy immobility variable is constructed for each year.

We use the operationalization of job lock provided by Huysse-Gaytandjieva et al. [9], which is based on results reported by Hanisch [4], that the average time thinking about quitting is one year. Employee is in job lock if he/she is dissatisfied with his/her job for two subsequent years and at the same time he/she stays in the same job. Correspondingly, the employee is in a long-term job lock if he/she continues to be dissatisfied with his/her job for more than two subsequent years and at the same time stays in the same job.

The job-dissatisfaction and job-immobility variables described previously, as well as the operational definition of job lock, are used to construct two nominal dependent variables for our analysis to present transitions to and from a job-lock state, respectively,

- transitions *to* the job lock state: 1 = dissatisfied and immobile in the preceding year and remaining in this state for two subsequent years (thus, prolonged job lock); 2 = mobile but dissatisfied with the job in the year before the job-lock state; 3 = immobile but satisfied with the job in the year before the job-lock state; 4 = mobile and satisfied with the job in the year before the job-lock state;
- transitions *from* the job lock state: 1 = dissatisfied and immobile for two subsequent year and remaining in this state during the next year (thus, prolonged job lock); 2 = mobile but still dissatisfied with the job in the year after the job lock state; 3 = immobile but satisfied with the job in the year after the job lock state; 4 = mobile and satisfied with the job in the year after the job lock state.

**Figure 1:**
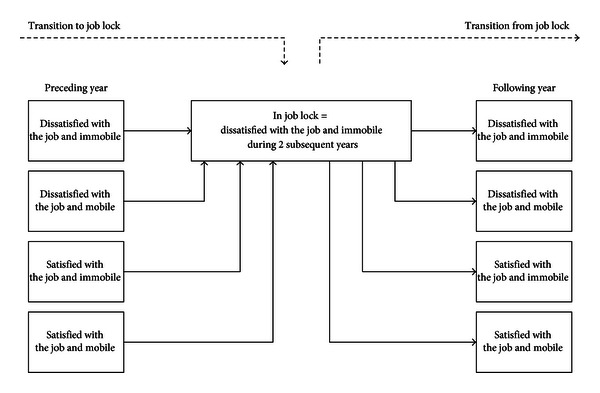
Transition models.

The two transition models are schematically presented in Figure 1. These are first-order Markov models where the probabilities of the values of the next state depend on the first order, thus on the previous state. Employees move through the two states according to four transitions per model as depicted in Figure 1 and as defined by the two nominal variables described previously. The movement of employees among states over time is tracked by transition probabilities. Hausman tests are run and show no dependence between categories of the dependent variables, which proves that the odds are independent of other alternatives.

We also define one binary dependent variable to compare those in a job-lock state (dissatisfied and immobile for two subsequent years, coded with 1) to those who are dissatisfied with their job but remain mobile during at least one of the years (not in a job lock situation even though job dissatisfied for two subsequent years, coded with 0). This way we include in our analysis all employees, who reported job dissatisfaction for two subsequent years (prolonged job dissatisfaction). The rest of the employees are omitted from the analysis.

### 3.3. Operationalization of the Explanatory Variables

The explanatory variables for our analysis represent six groups of factors that previous (economics and psychology) studies indicate as relevant in analyzing the state of job lock or its absence [9]. These groups of factors include not only sociodemographic, employment, occupational, sectoral, and contextual factors but also personality characteristics. As mentioned at the outset of this paper, we specifically focus on self-esteem and procrastination as personality characteristics that can explain the transition to and from a job-lock situation or the absence of such transition.

We use the response to the following question as an indicator of self-esteem: “Have you recently been thinking of yourself as a worthless person?” (0 = high, stable self-esteem; 1 = unstable, low self-esteem). The question is taken from the General Health Questionnaire (GHQ) included in the BHPS. The GHQ has been validated in nine countries [58] and has been used in various studies [59]. The fact that the above question requires a self-reported evaluation suggests that the answers to this question indicate explicit self-esteem. We apply the peak-end rule to construct the self-esteem variable for our analysis. The peak-end rule assumes that the value of an item (in this case, explicit self-esteem) which is measured at various points of time should not be represented by a simple average of all single evaluations. The value of that item can be better presented as a simple average of the peak—the most extreme value measured during the period—and the end—the value measured near the end of the period—that is, the peak-end value [60]. Following this rule, we construct the variable peak-end explicit self-esteem (called further on, self-esteem), which we use in the analysis. The application of the peak-end rule allows us to correct for eventual memory selectivity [60].

None of the existing measures of procrastination are directly applicable to work-related behavior [10]. The BHPS provides us with a proxy to measure procrastination: “Have you recently felt capable of making decisions about things?”. Based on this BHPS variable, we construct a dummy variable for our analysis that indicates procrastination (coded by 1) or the absence of it (coded by 0). This variable indicates decisional procrastination, but as indicated earlier in this paper, decisional procrastination and behavior procrastination are highly related [40]. Also, despite the temporal wording of the question, we use repetitive measure and in this way, we account for this weakness. Moreover, the question is not specifically related to work environment but indicates procrastination in the predecisional phase in general.

Health problems related to anxiety, depression, and so forth (“Do you have any of the health problems or disabilities: anxiety, depression or bad nerves, psychiatric problems”) are constructed as a dummy variable (coded: 0 = absence; 1 = presence). Age is measured as a continuous variable. The variables gender, marital status, working full time, member of the trade union, opportunities for promotion in the current job, belonging to the employer's pension scheme, and training as a part of the present employment are included in the analyses as dummies. Occupation is measured by the standard occupational classification (SOC). Nine dummy variables are included for occupation. Further, type of sector is included in the analysis as four dummy variables.

For an easier interpretation of the regression results, Table 5 presents the coding of all dummy variables used in the analysis.

### 3.4. Data Analysis

Bivariate correlation analysis provides supporting information to design the model [61], that is, information on the relevant variables to include in the model. Multinominal logistic regression is used to estimate the parameters of the two transition models: transition to and from a job-lock state. Thus, two multinominal logistic regressions are run separately. The baseline category in each regression analysis is the state of prolonged job lock (i.e., dissatisfaction and immobility for three subsequent years). In addition, binary regression is carried out to compare those in a job-lock state (dissatisfied and immobile for two subsequent years) to those who are dissatisfied with their job and mobile during at least one of the years (i.e. trying to adapt). For the sake of comparability, the set of explanatory variables remains the same across the models.

## 4. Results

Table 2 presents the results of the binary regression that analyses the differences between respondents who are in job lock (i.e., job dissatisfied and immobile during the two years) and those who are dissatisfied but mobile during at least one of the years. In total, 2949 employees report dissatisfaction with their job for two subsequent years and therefore are included in our analysis (see Table 1).

Of these, 1344 respondents experience job lock (i.e., they remain immobile during the two years) and 1605 respondents are mobile during at least one of the years. As the regression results suggest (see Table 2), the two groups differ significantly in terms of sociodemographic, employment, occupational, and sectoral factors (see coding of dummy variables in Table 5). In particular, among those dissatisfied with their job for two subsequent years, job lock is more often observed among elderly, married, men, and those with poor health, as well as among those in craft occupation, working full time, with an employer-provided pension scheme and without promotion opportunities. At the same time, job lock is less often observed among those with a managerial occupation. We do not find significant differences between the two groups with regard to the personality characteristics included in the analysis (peak-end self-esteem and procrastination), as well as with regard to contextual factors (i.e., regional unemployment rate).

As much as 61.4% of those who experience job lock (see Table 1) were dissatisfied and immobile during the preceding year (thus, they experience prolonged job lock). The other employees, who enter the job-lock state, were most often satisfied and immobile in the preceding year (26.1%). From those who are in job lock, 44.1% remains dissatisfied and immobile in the following year. Those who exit from the job lock state most often move to the “satisfied and immobile” state (32.4%) or “satisfied and mobile” state (14.1%).

Table 3 presents the results of the multinominal logistic regression. The first three columns of the table present the transitions from the job lock state to dissatisfied and mobile, satisfied and immobile, and satisfied and mobile states, respectively. The last three columns present the transitions to a job-lock state. The reference category for both models is dissatisfied and immobile. The coding of the dummy variables is presented in Table 5.  Table 4 highlights the results of the interaction terms included in the subsequent analysis. Specifically, the table presents the scores of the interaction terms separately for different extended versions of the models. In the following, we summarize the main results for the transitions to and from the job-lock state.

### 4.1. Transitions *to* a Job-Lock State

As indicated in Table 3, the transition to job lock from “dissatisfied and immobile” state (thus, prolonged job lock) is associated with a high regional unemployment rate. The transition to job lock from a state characterized by job satisfaction is associated with being in managerial, associate, or personal and protective service occupation as well as with low peak-end self-esteem and a tendency to decisionally procrastinate. In case of the “satisfied and mobile” state, the transition to job lock is also associated with sales and operative (plant and machine) occupations. This transition is also negatively related to age, government, or private sector. The transition to job lock from “dissatisfaction and mobility” state is negatively related to being married and having a craft occupation. The employees in personal and protective service occupation and those working in the private sector are more likely to transit to job lock from “dissatisfied and mobile” state. Our analysis shows no significant effect for variables indicating employment conditions. In addition to this, the interaction between peak-end self-esteem and regional unemployment rate appears significant (see Table 4). Thus, when regional unemployment rate is high, respondents with low peak-end self-esteem employees are more often “satisfied and immobile” than “dissatisfied and immobile.”

### 4.2. Transitions *from* a Job-Lock State

As suggested by Table 3, the exit from job lock to any of the three states is associated with having promotion opportunities. A high regional unemployment rate decreases the chance that employee would transit to any other state than “dissatisfied and immobile” (thus, it increases the chances of prolonged job lock). The exit from job lock to a state characterized with job satisfaction is negatively associated with age and positively associated with a service occupation and the private sector. Managers in job lock often exit to “dissatisfied and mobile” state or “satisfied and immobile” state. Having an associate occupation is positively associated with exit to “satisfied and immobile” state and full-time job is negatively associated with exiting to “satisfied and mobile” state. The probability to exit from job lock to a state characterized by job satisfaction is lower for those with low peak-end self-esteem.

Four interactions show a significant effect. First is the interaction between peak-end self-esteem and health for the transition from “satisfied and mobile.” At the same time the effect of peak-end self-esteem variable becomes not significant while the effect of the health variable does not change. Second is the interaction between procrastination and health. This changes the effect of procrastination to become significant while keeping the effect of the health variable. Third is the interaction between age and peak-end self-esteem. This changes the effect of self-esteem to insignificant while keeping the effect of age. Fourth is the interaction between peak-end self-esteem and the regional unemployment rate.

Additionally, we checked how many employees stay dissatisfied and immobile for 4 and 5 subsequent years. They are 136 and 47, respectively, which shows a decreasing trend.

### 4.3. Summary of Main Findings

Overall the results show that being older, being married, working in a craft occupation, in the governmental sector, having a full-time job and high regional unemployment rate increase the likelihood that an employee enters the long-term job lock state. Furthermore, low peak-end self-esteem, mental health problems, and decisional procrastination show significant effects on the probability to stay in long-term job lock (failure to exit the job-lock state). On the contrary, having a managerial, service, or associate occupation, working in the private sector, and having promotion opportunities increase the chance of an exit from the state of job lock. A high regional unemployment rate is not statistically significant for those dissatisfied in two consequent years. It seems that a high regional unemployment rate provides incentives for employees who are dissatisfied to adapt by adjusting. Among dissatisfied employees, older workers are less likely to use mobility as an adaptation strategy. Further, possessing a company pension scheme increases the likelihood that the employee, who is dissatisfied, is immobile for two subsequent years.

## 5. Discussion

Our results highlight the process of the transition to and from a job-lock situation, as well as the situation of long-term job lock. We briefly discuss the key findings in the subsequent paragraphs.

### 5.1. What Factors Push Employees in a Job-Lock State?

Our results suggest that being older, married, with low peak-end self-esteem, working in a craft occupation, in the governmental sector, and high regional unemployment rate are push factors to a job-lock state.

As previous research also shows, elderly employees are less mobile [62, 63]. This can be explained by the fact that growing older is related to higher job investments made. However, preceding studies also show that elderly employees are more often job satisfied. Additionally, Clark et al. [64] showed that job satisfaction is U-shaped in age. In other words, employees at the beginning and end of their career are more inclined to experience satisfaction and those in the middle age are more often dissatisfied. Nevertheless, we find that age is more likely to be related to job lock. This can be explained by the fact that perceived control may diminish with age [24, 65] and, in turn, this may lead to diminished use of problem-focused coping and as a result affects well-being [66].

Another important variable concerning mobility is marital status. Being married is negatively correlated with the probability of quitting when dissatisfied with the job [67]. Married employees are also less satisfied with their job than single individuals [68] and more often fall in job lock. This situation is observed irrespective of previous findings that married people are in general more satisfied and happier. Based on the spillover theory, however, one can expect that marital satisfaction would affect work satisfaction. Thought as previous studies have shown, the two variables not always change together—the changes in one domain do not fully match the changes in other areas [69]. Thus, it might be easier for an individual to accept dissatisfaction if it is related just to a certain domain (job dissatisfaction) when there is satisfaction in the other areas of life (marital state).

Employment, occupational, sectoral, and contextual factors also may push individuals to a job-lock state. As previous studies have shown when the regional unemployment rate increases, employees are more likely to be immobile [70]. Furthermore, the high regional unemployment rates may lessen overall satisfaction by simply diminishing the supply of labor opportunities. In support of the information about the trends in public sector employment in the UK (particularly the steady decline for those working for local governments), we found that those working in the governmental sector are more likely to be both job dissatisfied [71] and immobile [72]. This has been explained by increased workloads and stress in this sector. Also we find that being in a craft occupation increases the likelihood that the employee would be “dissatisfied and immobile” for a longer period of time (i.e., higher chance to enter prolonged “job lock”). This might be explained by the specific skilled work that craft occupations require and the often small-scale production of goods which may hinder both internal and external mobility when dissatisfied.

### 5.2. Why Do Some Employees Fail to Exit the Job Lock?

The factors that push an employee into job lock—age, low peak-end self-esteem, and high regional unemployment rate—also play an essential role in the failure to exit job lock and enter long-term job lock. In addition to the already discussed variables, having a full-time contract increases the probability to enter the long-term “job lock” state rather than to move to any of the other states. This confirms the outcome of previous studies that full-time workers are less mobile [73] and less satisfied [74]. Besides, our study outlines tendencies (traits) to stay dissatisfied and immobile for full-time employees. This can be explained by the importance of work to those who work full-time. Additionally, it can be that commitment and job investments are higher for those working full-time compared with others in parttime work.

### 5.3. What Factors Are Associated with the Exit from the Job-Lock State: Pull Factors?

Some occupations play a significant role in the transitions from “job-lock” state. In particular, being in a managerial or administrator position, associate professional, personal and protective service increases the chance that the employee exits the “job-lock” state. Further, private sector and promotion opportunities pull employees out of job lock. Holding a personal and protective occupation increases the chance that an employee moves to “satisfied and immobile” or “satisfied and mobile” instead of remaining in a job-lock state.

Individual abilities play an important role in occupational decision making [75]. Furthermore, every occupation requires certain skills that the employee can have a match with or not [76]. It might be that career achievement brings not only promotion possibilities but also satisfaction [77]. Moreover, we may expect that career stages (exploration, establishment, midcareer, late career, and decline) [77] are part of every occupation. Nevertheless, the duration of every stage would be different for different occupations which together with the individual importance of work and career can influence the transitions.

Besides, having promotion opportunities in the current job increases the likelihood that the employee moves to one of the other three states. In general having promotion opportunities in the current job increases overall job satisfaction [78]. And, promotion opportunities are themselves mobility opportunities, that is, internal mobility.

### 5.4. Mobility or Adaptation: What Coping Strategy Can Help to Exit a Job-Lock State?

Employees in a manager and administrator occupation, those in personal and protective service occupations, with promotion opportunities, working in the private sector use much more often mobility as a coping strategy in order to adjust to job dissatisfaction.

Thus, managers and administrators and personal and protective service occupations are capable of successfully adapting to job dissatisfaction by using active forms of adaptation. It might be that for employees in those occupations, job dissatisfaction is just one of the drivers for job mobility. Qualities to succeed in your job may be also essential qualities for successful adaptation.

At the same time, manager and administrator occupation, associate professional, personal and protective service occupations, sales, working in the private sector, and with promotion opportunities, employ work adjustments as coping strategy (satisfied and immobile).

Thus, almost the same variables play a role in successful adaptation independent from the form of the adaptive strategy (either with job satisfaction or job mobility). We can conclude that people are either capable of successfully adapting or not irrespective of the coping strategy used.

### 5.5. The Relevance of Self-Esteem and Procrastination in Research on Job Lock

Peak-end low self-esteem for both multinominal models is related to the transition to job lock or long-term “job lock.” On the other hand, the analysis of employees who are dissatisfied for two years does not show self-esteem to be significant compared to those who moved and those who stay in a job lock. Further, the findings support our expectation that the inclusion of the procrastination and psychological health variables in our model leads to a better explanation of long-term “job lock.” Peak-end low self-esteem, mental health problems, and decisional procrastination show significant effects on the probability to stay in long-term “job lock.” Low self-esteem plays a role in failure to adapt to job dissatisfaction. However, having high self-esteem does not show guarantee for successful adaptation.

Low self-esteem and mental health problems have a joint effect on the transition from a job-lock state (shown by the significance of the interaction term). This is in line with the results from previous research that depression and anxiety are some of the symptoms experienced by people with low self-esteem [79, 80]. Positive self-evaluations are vital for psychological health [81].

Additionally, the analysis comparing coping strategies by dissatisfied employees shows that those with poor health are much more often mobile. It might be that health deterioration leads to an adjusted job, internal mobility, or what some other studies show—the severer the mental health the sooner the employee leaves [82]. Thus mental health problems might be related to impulsivity (impulse to quit when job dissatisfied). Additionally, we may expect that health problems bring general dissatisfaction. We may expect that mental health diminishes the quality of life and influences overall happiness [83]. Nevertheless, the results of our study show that mental health has no separate effect on prolonged job lock or quitting job lock.

Due to the fact that procrastination has been seen as a risk factor for more serious depression and anxiety [84], we include an interaction term between mental health problems and procrastination in our model. Decisional procrastination and mental health problems show dependency. Employees who procrastinate to make decisions and report mental health problems are less likely to be “satisfied and mobile” compared to “dissatisfied and immobile” and become stuck at their job in the long term (i.e., to experience job lock). While decisional procrastination is shown to be preventing employee from entering a job lock state, procrastination proves to be much more related to job satisfaction. One explanation for that is that in the short run, it plays the role of an adapting mechanism. However previous research shows that when procrastination is used in this way, it simply adds additional stress and becomes very inadequate [85]. Thus, decisional procrastination is a maladaptive coping mechanism for handling conflicts in decision making [39].

We find that when regional unemployment rate is high, employees with low self-esteem have less chance to experience job dissatisfaction. It might be that when there are fewer opportunities on the labor market, people see their present job in a more favorable light and report more satisfaction. Employees may also realize that they are happy to have a job, or it could be a selection effect where dissatisfied employees are more likely to be laid off. Additionally, this employees' behavior might be influenced not by the actual job availability but by their perceptions of job availability [30].

## 6. Conclusion

The aim of this study has been to explore the process of the transition to and from a job-lock situation, as well as the situation of long-term job lock. Our results provide insights into understanding individual differences in adaptation which help to illuminate when and why successful adaptation does or does not occur.

Another contribution of the present study is that by following the process—from dissatisfaction to job lock to long-term job lock—we are able to distinguish the essential variables which play a role in the state transitions. Further, we faced Diener et al.'s [69] challenge to differentiate passive versus active coping in adaptation. The present study offers an understanding of the process of long-term “job lock.” Further, by bringing light to the adaptation process we provide information that is useful for designing successful interventions. Our findings contribute to the field of adaptation to job dissatisfaction and the limited research in the area of procrastination at work.

Nevertheless, the current findings should be interpreted with caution because of the following limitations of the present study: lack of differentiation of voluntary and involuntary job mobility, mental health problems, and the usage of a proxy measure of self-esteem and procrastination. Nonetheless, none of the existing measures of trait procrastination are directly appropriate to work-related behavior [10]. Future study is needed to develop a measurement instrument for procrastination at work. Further, it is important to distinguish between different types of procrastinators and find out which one is related to long-term job lock. Procrastination might be more complex than merely related to stress, mental health, and hindrance of performance [86]. It can be that procrastination is not always dysfunctional. In some cases, procrastination behavior might lead to positive outcomes, such as a lower level of stress and depression and greater life satisfaction [87, 88]. Thus, there might be more than one kind of procrastinators. Several studies show that procrastinators are not a homogenous group and there are certain types of procrastinators who might be more prone to emotional problems [44]: such as anxiety [89], arousal and avoidance motives [90], optimistic and pessimistic [91], and passive and active type [87]. Further, people may have a need to procrastinate in one area (work, relationships, insurance, etc.) more than in other areas. Further, the results of this study concern adaptation to job dissatisfaction and might be not applicable to adaptation in other areas of life. The extent of adaptation varies for different life events [69].

Irrespective of the study limitations, the results can be applied on different levels: at the individual, organizational (HRM, company doctors, managers, coaches, mental health professionals), and societal levels (labor unions, government) by increasing awareness and knowledge, using them for prevention, in problem solving, and development of supportive programs.

In terms of practical implications, the current findings present a need to develop and incorporate programs tackling task avoidance and procrastination at the work place. Additionally, counseling to support employees with dysfunctional procrastination tendency can be beneficial as employee's stress reduction may increase productivity. The results might be beneficial to those who are involved in employee selection and those who are responsible for making promotion decisions.

## Figures and Tables

**Table 1:** Descriptive statistics.

Job dissatisfaction during two subsequent years and …	Frequency		Transition from … to job lock	Frequency		Transition from job lock to …	Frequency	
	*N *	%		*N *	%		*N *	%
0 = mobile during at least one of the years (not in job lock)	1605	54.4	1 = dissatisfied and immobile	484	61.4	1 = dissatisfied and immobile	364	44.1
			2 = dissatisfied and mobile	51	6.5	2 = dissatisfied and mobile	78	9.5

1 = immobile during both years (in job lock)	1344	45.6	3 = satisfied and immobile	206	26.1	3 = satisfied and immobile	267	32.4
			4 = satisfied and mobile	47	6.0	4 = satisfied and mobile	116	14.1

Total	2949	100		788	100		825	100

**Table 2:** Results of binary probit regression.

Explanatory variable	Job dissatisfaction for two subsequent years		
	0 = not in job lock (i.e., mobile during at least one of the years)		
	1 = in job lock (i.e., immobile during both years)		
	Coefficient	Std. error	Odds ratio
Sociodemographic features			
Age	0.024**	0.003	1.035
Gender	0.139*	0.058	1.261
Health status	−0.419**	0.106	0.505
Marital status	0.127*	0.057	1.234
Personality attributes:			
Min peak-end self-esteem	0.054	0.073	1.076
Procrastination	−0.049	0.084	0.932
Type of occupation (reference category: clerical and secretarial)			
Manager and administrators	−0.369**	0.097	0.545
Professional	0.030	0.100	0.946
Associate professional/technical	−0.084	0.105	0.875
Craft	0.448**	0.094	2.093
Personal and protective service	−0.203	0.108	0.718
Sales	−0.064	0.116	0.891
Plant and machine	0.095	0.088	1.171
Other occupations	0.079	0.110	1.167
Employment conditions			
Full-time contract	0.360**	0.087	1.794
Employer pension scheme	0.318**	0.076	1.677
Member of the trade unions	−0.035	0.082	0.949
On-the-job training	0.071	0.055	1.128
Promotion opportunities	−0.627**	0.068	0.355
Type of sector (reference category: army and other sectors)			
Civil	−0.388**	0.141	0.524
Governmental	−0.580**	0.115	0.382
NHS or higher education	−0.867**	0.142	0.236
National industry	0.099	0.207	1.137
Nonprofit	−0.859**	0.213	0.239
Private	−0.928**	0.085	0.214
Work-related contextual features			
Regional unemployment rate	−0.005	0.013	0.995
Intercept	−0.123	0.220	
Observations	2949		
Pseudo *R* ^2^	0.147		

*Significant at 5% level; **significant at 1% level.

**Table 3:** Results of multinominal logistic regression.

Explanatory variables	Transition from job lock to …: (reference category: dissatisfied and immobile)			Transition from … to job lock: (reference category: dissatisfied and immobile)		
	Dissatisfied and mobile	Satisfied and immobile	Satisfied and mobile	Dissatisfied and mobile	Satisfied and immobile	Satisfied and mobile
	Odds ratio	Odds ratio	Odds ratio	Odds ratio	Odds ratio	Odds ratio
Sociodemographic features						
Age	0.977	0.981*	0.933**	1.008	0.989	0.928**
Gender	1.298	0.698	0.577	0.751	0.764	0.662
Marital status	0.600	0.905	0.846	0.405**	0.743	0.496
Health problems: anxiety, depression, and so forth	2.129	0.814	1.457	0.499	0.941	0.447

Personality attributes:						
Min peak-end self-esteem	0.689	0.409**	0.413*	0.685	0.557*	0.121**
Procrastination	1.137	1.149	1.436	0.609	1.982*	3.259*

Type of occupation (reference category: clerical and secretarial):						
Manager and administrators	2.974*	2.285*	2.185	1.976	3.371**	12.505**
Professional	0.870	1.841	2.109	2.404	1.686	8.758**
Associate professional/technical	2.713	3.805**	2.652	0.473	2.989**	7.848*
Craft	0.342	1.521	0.913	0.100*	1.837*	3.725
Personal and protective service	1.563	3.381**	4.110*	7.748**	3.361**	3.215
Sales	1.820	0.905	1.947	1.445	1.560	7.070*
Plant and machine	0.296	0.925	0.396	0.778	1.171	6.499**
Other occupations	0.706	1.465	0.580	2.086	1.712	1.903

Type of sector: (reference category: other sectors):						
Private	1.899	1.977*	3.112**	3.909*	1.114	0.225**
Civil	1.731	0.999	1.145	2.002	0.631	1.394
Governmental	1.239	1.346	0.907	0.847	1.293	0.276*

Employment conditions:						
Full-time contract	0.430	0.627	0.129**	1.447	0.735	0.386
Employer pension scheme	1.796	1.349	1.811	0.832	1.508	0.660
On-job training	0.984	1.132	0.764	1.414	0.923	0.685
Promotion opportunities	3.991**	2.329**	14.572**	0.829	0.985	0.976
Member of the trade unions	0.532	0.667	0.608	0.546	0.968	1.636

Work-related contextual features:						
Regional unemployment rate	0.836**	0.864**	0.818**	0.857*	0.794**	0.828*

Other model characteristics	Observations = 825			Observations = 782		
	LR *χ* ^2^ (df = 69) = 287.25			LR *χ* ^2^ (df = 69) = 192.43		
	Pseudo *R* ^2^= 0.1437			Pseudo *R* ^2^ = 0.1238		

*Significant at 5% level; **significant at 1% level.

**Table 4:** Interaction terms.

Variables/interactions	Satisfied and mobile
Dependent variable: transition from job lock to … (reference: dissatisfied and immobile)	

Peak-end self-esteem ∗mental health problems	0.076*
Peak-end self-esteem	3.944
Mental health problems	3.114

Mental health problems ∗ procrastination	0.088*
Mental health problems	2.643
Procrastination	10.680*

Age ∗ peak-end self-esteem	0.897*
Peak-end self-esteem	19.341
Age	0.942**

Regional unemployment rate ∗ peak-end self-esteem	1.461*
Peak-end self-esteem	0.014**
Regional unemployment rate	0.774**

	Satisfied and immobile

Dependent variable: transition from … to job lock (reference: stay dissatisfied and immobile)	

Regional unemployment rate ∗ peak-end self-esteem	1.361*
Peak-end self-esteem	0.033**
Regional unemployment rate	0.759**

*Significant at 5% level; **significant at 1% level.

**Table 5:** Coding of the dummy variables used in the analysis.

Dummy variables	Dummy codes	
	0	1
Gender	Female	Male
Marital status	Separated; divorced; widowed; never married	Married
Health problems: anxiety, depression, and so forth	No	Anxiety, depression, or bad nerves
Decisional procrastination	More than usual; same as usual	Less so; much less
Self-worth Private sector Civil sector Governmental sector	Not at all; no more than usual No No No	Rather more; much more Yes Yes Yes
Full-time contract Employer pension scheme On-job training Promotion opportunities Member of the trade unions	No No No No No	Yes Yes Yes Yes Yes
